# Mass mortality of the invasive alien echinoid *Diadema setosum* (Echinoidea: Diadematidae) in the Mediterranean Sea

**DOI:** 10.1098/rsos.230251

**Published:** 2023-05-24

**Authors:** Rotem Zirler, Lisa-Maria Schmidt, Lachan Roth, Maria Corsini-Foka, Konstantinos Kalaentzis, Gerasimos Kondylatos, Dimitris Mavrouleas, Emmanouil Bardanis, Omri Bronstein

**Affiliations:** ^1^ School of Zoology, Faculty of Life Sciences, Tel Aviv University, Israel; ^2^ The Steinhardt Museum of Natural History, Tel Aviv University, Israel; ^3^ The Interuniversity Institute for Marine Science in Eilat, Eilat 8810302, Israel; ^4^ Hydrobiological Station of Rhodes, Hellenic Centre for Marine Research, Cos Street, 85100 Rhodes, Greece; ^5^ Naturalis Biodiversity Center, PO Box 9517, 2300 RA Leiden, The Netherlands; ^6^ Department of Biology, National and Kapodistrian University of Athens, 15784 Athens, Greece

**Keywords:** *Diadema*, mass mortality event, Levantine Basin, tropicalization, alien species, pathogens

## Abstract

The sea urchin *Diadema setosum* is an ecological key species across its range, particularly on coral reefs. In 2006 *D. setosum* was first observed in the Mediterranean Sea, and since, it has proliferated to occupy the entire Levantine Basin. Here we report the mass mortality of the invasive *D. setosum* in the Mediterranean Sea. This is the first report of *D. setosum* mass mortality. The mortality spans over 1000 km along the Levantine coast of Greece and Turkey. The current mortality shows similar pathologies to previously reported *Diadema* mass mortality events, suggesting pathogenic infection as the cause of mortalities. Maritime transport, local currents, and fish predation of infected individuals may distribute pathogens at varying geographical scales. Due to the proximity of the Levantine Basin to the Red Sea, the risk of pathogen transport to the native Red Sea *D. setosum* population is imminent—with potentially catastrophic consequences.

## 1. Background

Echinoids are widely recognized as the single most important group of consumers in shallow marine habitats [1]. As grazers, they restrict algal growth, clearing up space for larval settlement and restraining algal proliferation that may outcompete slower growing organisms, such as corals [2]. However, at high abundances, echinoid grazing may cause extensive bio-erosion of hard benthic substrates, gradually leading to degradation of benthic habitats and ultimately loss of biodiversity [3]. Nevertheless, despite their potential large-scale impact, relatively few echinoid taxa functionally control the structure and function of a wide range of marine communities [1].

The most abundant and ecologically significant shallow-water echinoids to date are members of the genus *Diadema* Gray, 1825 [3]. Extant species include *Diadema palmeri* Baker, 1967 and *Diadema mexicanum* A. Agassiz, 1863 from New Zealand and the eastern Pacific, respectively [4,5]. The Atlantic is inhabited by *Diadema antillarum* (Philippi, 1845) in the west, and *Diadema africanum* Rodríguez, Hernández, Clemente & Coppard, 2013 in the east [4–6]. *Diadema clarki* Ikeda, 1939, occurs in southern Japan [4]. The three Indo-Pacific *Diadema* are: *Diadema paucispinum* A. Agassiz, 1863 (also found in the South Pacific), *Diadema savignyi* (Audouin, 1809) and *Diadema setosum* (Leske, 1778) [4,5]. *D. setosum* consists of two genetic clades: the widely distributed clade a, ranging from the western Pacific and east coast of Africa [4,5], and clade b, originally confined to the Red Sea (RS) and Persian Gulf [4,5,7], and recently invading to the Mediterranean Sea [8,9].

In 2006 *D. setosum* was observed for the first time in the Mediterranean Sea, off Kas¸ peninsula, Turkey [9]. Since then, it has expanded its range throughout the coasts of Greece [10], Lebanon [11], Israel [8], and most recently Egypt [12] and Libya [12], marking the species' occupation of the entire Levantine Basin and neighbouring seas (Aegean and Ionian) [10,13].

Apart from the natural connection to the Atlantic Ocean in the west, the Mediterranean was recently connected to the RS through the man-made Suez Canal [14]. The canal opened in 1869, forming a maritime highway for tropical RS species migration to the Mediterranean [15]—termed *Lessepsian Migration* [16]. As global change proceeds, tropicalization of the eastern Mediterranean intensifies, facilitating the proliferation of warm-water alien species—leading to competitive exclusion and biodiversity loss of native biota [14,17].

**Figure 1.**
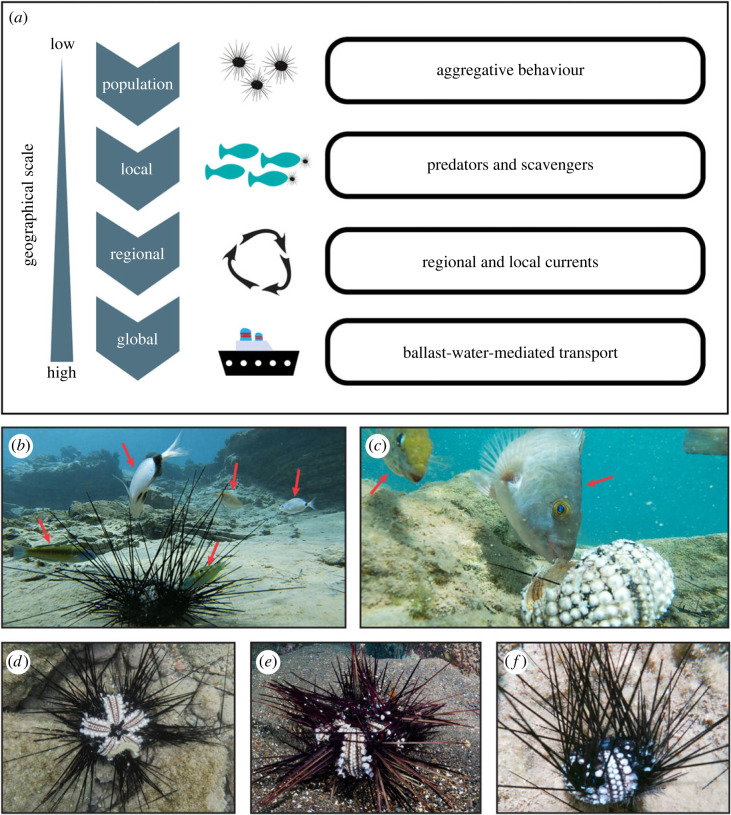
*Diadema* mortality. (*a*) Geographical scales of *Diadema* pathogen transport during mass mortality events. (*b,c*) Scavenger fish (red arrows, from left to right: *Thalassoma pavo*, *Diplodus vulgaris*, *Thalassoma pavo*, *Siganus rivulatus*, *Diplodus annularis*, *Siganus rivulatus*, and *Sparisoma cretense*) feeding on dead and dying *D. setosum* at Alimia Island on 13 September 2022. (*d*) *D. antillarum* (credit: K. Kitson-Walters), (*e*) *D. africanum* (credit: João Monteiro) and (*f*) *D. setosum* exhibiting typical tissue loss associated with pathogenic driven mortality.

Mass mortality events (MMEs) refer to a sudden and dramatic reduction in the number of individuals of a given population, following extensive mortality [18,19]. One of the best-studied MMEs is the population collapse of *D. antillarum* in the Caribbean and western Atlantic. Reoccurring die-offs of *D. antillarum* with peaks in the early 1980s and recently in 2022, caused by a waterborne pathogen [20], have led to dramatic phase shifts in benthic communities; from thriving coral reefs to an algal-dominated state [21–24]. Evidence of mass mortalities from the eastern Atlantic, affecting the closely related species *D. africanum*, first appeared in 2009 (figure 1*e*) [25], showing highly similar pathologies (e.g. epidermal necrosis and typical spine loss) to the ones formerly observed in *D. antillarum* (figure 1*d*). *Vibrio alginolyticus*, a waterborne bacterium, in combination with anomalous high seawater temperatures, was identified as the main driver of *D. africanum*'s 2009 MME in East Africa [26]. Following this MME, *D. africanum* populations were reduced by 65%, and during a subsequent MME in 2018, the local population collapsed by 93% in the Canary Islands [27] promoting a stable shift toward a macroalgae-dominated system [28]. Also in 2009, and in contrast to the earlier extensive *D. antillarum* MME in the Caribbean [29], localized mortalities of *Diadema mexicanum* were reported from the eastern Pacific, and attributed to pathogenic infection [30]. However, the latter mortality was limited to a single reef in Mexico and thus could not be viewed as mass mortality. Here we present first evidence of an ongoing mass mortality of *D. setosum* in the Mediterranean Sea (figure 1*f*). This is the first report of *D. setosum*'s MME and the only known report of an invasive species mortality in the Mediterranean Sea. Mostly occurring around the coasts of Greece and Turkey, these die-offs are currently concentrated near the Island of Rhodes and Kas¸ peninsula. We compiled reports from both scientific and citizen sources, analysed the geographical locations of reported mortalities, and investigated the symptoms and pathologies of dead and dying individuals *in situ*. Finally, we compare the current Mediterranean mortality to other reported mortalities of *D. antillarum* and *D. africanum*, discuss potential drivers and means of transmission of the observed symptoms, and evaluate the potential consequences of this mortality on both local and regional scales.

## 2. Methods

Data on abundance and health of *Diadema* were obtained by visual surveys while snorkeling down to 5 m depth. Additional observations were collected from citizens involved in daily sea activity in their respective regions (i.e. local fishermen and diving instructors).

**Figure 2.**
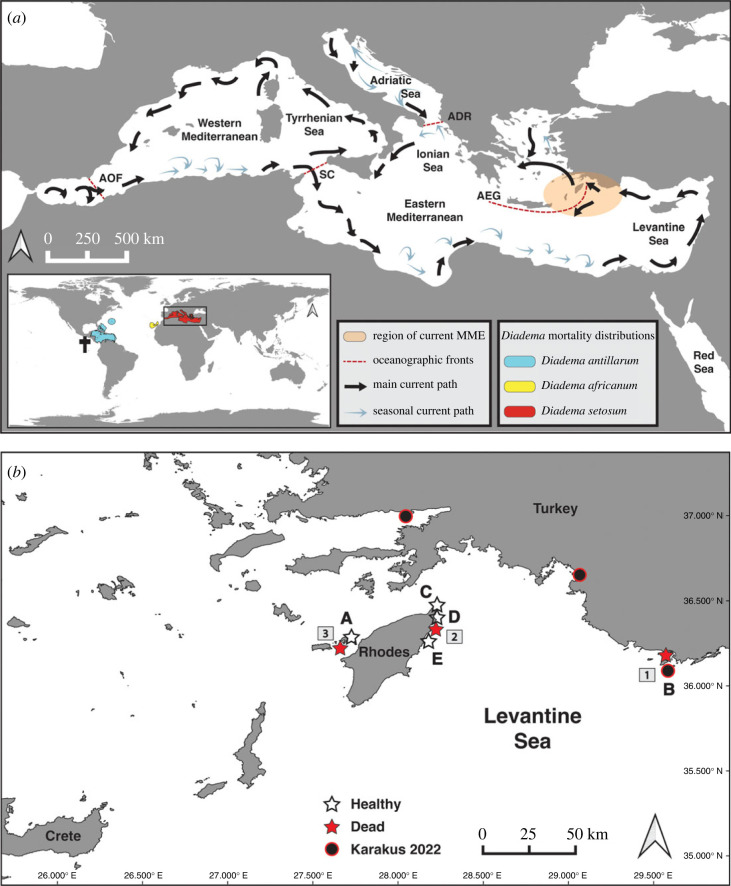
Maps of historical and current *Diadema* mass mortality events. (*a*) The Mediterranean Sea, divided into regions separated by marine natural barriers (dashed red lines). Main and seasonal currents are represented by black and blue arrows, respectively [31]. Bottom left: global scale view of *Diadema* mass mortalities: western Atlantic Ocean (blue), eastern Atlantic Ocean (yellow) and Mediterranean Sea (red). Black cross indicates the location of localized *D. mexicanum* mortality [30]. AOF, Almeria-Oran Front; SC, Sicily Channel; ADR, Otranto Channel; AEG, southern Aegean boundary. (*b*) Locations of healthy (white stars) and dead (red stars) *D. setosum* within the Levantine Basin of the Mediterranean Sea. Black circles indicate mortality locations reported by Karakus [32]. Numbers indicate the temporal sequence of occurrences within the Mediterranean—indicating an east-to-west trajectory. Letters correspond to site names in table 1.

Surveys on Alimia Island (location A; figure 2*b*) were conducted on 10 July 2022 and 13 September 2022 at a depth range of 0–5 m. Healthy and dead/dying individuals were counted. An underwater camera was placed near dead and dying *Diadema* individuals to record fish species feeding on them. On both dates, notes on the population density and health of other, native sea urchins (i.e. *Arbacia lixula* (Linnaeus, 1758) and *Paracentrotus lividus* (Lamarck, 1816)) were taken.

**Table 1.** Healthy and dead Mediterranean *D. setosum* reported in the current study. Citizen science reports are denoted by asterisks and the corresponding population densities for these observations are given in three level scale estimates: + (greater than 5); ++ (5–20); +++ (less than 20).

date of observation	location (map reference)	coordinates	depth (m)	number of observed individuals	health state	symptoms	adjacent skeletal elements	adjacent echinoid species
10 Jul 2022	Alimia Island (A)	36.265278, 27.707778 through 36.268611, 27.708889	0–5	4–5 m^−2^	healthy	none		
15 Jul 2022*	Kastellorizo Harbour (B)	36.150044, 29.590793	0–5	++	dead	spine and tissue loss		
21 Jul 2022*	Anthony Quinn Bay, Rhodes (C)	36.321916, 28.210098	3–4	++	healthy	none		
22 Jul 2022ֿ*	Anthony Quinn Bay, Rhodes (C)	36.321916, 28.210098	3–4	+	dead	spine loss	exposed test	
1 Aug 2022*	Anthony Quinn Bay, Rhodes (C)	36.321916, 28.210098	NA	+++	dead and dying	spine loss	exposed test	
6 Sep 2022	St Nicholas Fortress, Rhodes (D)	36.451534, 28.227882	0–5	0.0006 m^−2^	healthy	none		
6 Sep 2022	Karakonero, Rhodes (E)	36.421454, 28.231733	0–5	0.0004 m^−2^	healthy	none		5–7 *Arbacia lixula* (healthy)
13 Sep 2022	Alimia Island (A)	36.265278, 27.707778 through 36.268611, 27.708889	0–5	4–5 m^−2^	dead	tissue loss, attached spines	scattered spines and Aristotle's lanterns	*Arbacia lixula* (healthy) *Paracentrotus lividus* (healthy)
13 Sep 2022	Alimia Island (A)	36.265278, 27.707778 through 36.268611, 27.708889	15–20	4–5 m^−2^	dead	spine and tissue loss		
15 Sep 2022	Anthony Quinn Bay, Rhodes (C)	36.321916, 28.210098	0–5	0.0005–0.0006 m^−2^	healthy	none		5–10 *Arbacia lixula* (healthy)

Records from Kastellorizo Harbour (location B; figure 2*b*) from 15 to 20 July 2022 were collected by snorkeling. Live or dead individuals were not counted; hence no quantifiable estimations could be obtained for this site.

Surveys at St Nicholas Fortress (location C; figure 2*b*; approximate surveyed area: 3500 m^2^) and Karakonero (locality D; figure 2*b*; approximate surveyed area: 2600 m^2^) were conducted on 6 August 2022. Individuals of *D. setosum* as well as indigenous sea urchin species (see above) were counted.

Surveys at Anthony Quinn Bay (location E; figure 2*b*; approximate surveyed area: 5000 m^2^) were conducted on 15 September 2022. Supplementary observational data from July were provided by a local diving instructor performing daily dives at this site (0–10 m).

## 3. Results and discussion

Dead and dying *D. setosum* were observed for the first time on 15 July 2022, offshore Kastellorizo Harbour (Greece) (table 1; figure 2*b*, area 1), adjacent to the coast of Kas¸ (Turkey)—the first location from where *D. setosum* was reported in the Mediterranean Sea. On 22 July, additional mortalities were discovered near Anthony Quinn Bay (Rhodes) (table 1; figure 2*b*, area 2) among healthy looking individuals at the same site. On 1 August, more than 100 dead and dying *Diadema* were recorded at the exact same site, with no apparent healthy individuals present. Mortalities continued through September at both shallow (0–5 m) and deeper (15–20 m) water near Alimia Island (table 1; figure 1*b*, area 3; electronic supplementary material, S1). Recently, observations of potential mortalities were also reported from the vicinity of Gökova and Fethiye in southern Turkey [32]—suggesting that the scope of the current Mediterranean MME extends over 1000 km. Consequently, the geographical scale of the current Mediterranean MME rules out the possibility that mortalities are caused by a local acute driver such as pollution. Recording mortalities at depths of 15–20 m demonstrates that the driver of fatalities is not restricted to shallow water where environmental effects are most potent. During these events, dead and dying individuals were reported to experience tissue and spine loss, and carcasses were observed on the seabed (electronic supplementary material, S1–S4). Interestingly, surveys at some of the sites, only several days prior to the reported MMEs, revealed no signs of illness (table 1)—indicating an abrupt effect of the drivers of mortality on a seemingly healthy population.

Our data show that *D. setosum* is currently undergoing mass mortality in the eastern Mediterranean Sea. To our knowledge, this is the first confirmed report of *D. setosum* MME—making it the third species in the genus to undergo extensive die-offs. Furthermore, while mass mortalities of native Mediterranean species have been previously discussed in the scientific literature (e.g. *Pinna nobilis* [33,34] and *Spongia officinalis* [35]), this is, to the best of our knowledge, the first report of an invasive alien species MME in this region. While linkage between reported *Diadema* mass mortality events (1983: western Atlantic, *D. antillarum* [21,24,29]; 2009: eastern Atlantic, *D. africanum* [26]; 2022: Mediterranean Sea, *D. setosum*) has not been established, identification of the potential pathogen in the Mediterranean may shed light on underlying global trend of this phenomenon.

As no specimens have currently been collected from the Mediterranean MMEs, unambiguous identification of the cause of mortality is impractical. Nevertheless, careful *in situ* observations as well as examination of footage from dead and dying individuals from all reported *Diadema* MMEs demonstrate markedly similar pathologies (figure 1; electronic supplementary material, S1–S4). Progression of the symptoms seem to be rapid, leading to death within two days. Initially, loss of tube feet hampers the ability of individuals to attach to the substrate, causing many to get swept off their niches and accumulate by local eddies. Spine movement becomes slow and less responsive. Shortly after, typical lesions appear in areas of tissue loss along the apical ambulacrum, and spines fall off leaving bare areas clearly visible (figure 1*b–f*). Finally, the sea urchin dies, leaving behind skeletal remains: empty tests, piles of spines, and scattered Aristotle lanterns. These pathologies seem to be unique and differ by appearance from other pathologies associated with echinoid mortalities—such as predation, osmotic stress or heat shock. Additionally, no storms or heavy rainfall were recorded in the days prior to the mortalities. Consequently, our current working hypothesis is that the ongoing MME in the Mediterranean is pathogen driven.

Observations of mortalities at 20 m depth may provide support for the notion that these MMEs are not directly related to elevated temperatures—a known driver of marine mass mortalities [36]. Furthermore, the native range of *D. setosum* clade b that inhabits the Mediterranean [37] includes the Persian Gulf [4,5], where water temperatures may exceed 36°C [38]—well beyond the temperatures recorded at the sites of mortalities. Still, elevated temperatures are known to accelerate marine pathogenic outbreaks [26,39,40] and the rapid warming of the eastern Mediterranean due to global change is expected to accelerate and intensify pathogen outbreaks in the region.

The effects of *Diadema* mortalities in the eastern Mediterranean may be substantial on both local and regional scales. Several indigenous shallow-water echinoids, such as *Arbacia lixula* and *Paracentrotus lividus*, are sympatric with *Diadema* in the Levant [41,42]. The two were previously reported to suffer similar symptoms to those currently displayed by *Diadema* [41,43]. Nonetheless, our surveys recorded the presence of healthy individuals of both species in proximity to dead and dying *Diadema*, presenting the opportunity of infection by a presumable pathogen (although current infections have not been detected) (table 1; electronic supplementary material, data S1d). Additionally, the eastern Mediterranean is also home to the endemic diadematoid *Centrostephanus longispinus* [44,45]. Although the overall depth ranges of *D. setosum* and *C. longispinus* differ, the two have been observed in sympatry off the Mediterranean coast of Israel (personal observation). This and the close genetic proximity between the two species may put the native *C. longispinus* at risk of infection.

The natural aggregating behaviour of *Diadema* [4] may accelerate the spread of potential pathogens within populations. Several Mediterranean fish species (table 2), native and alien, were documented interacting with dead and dying *D. setosum*, mostly through predation and scavenging of dead specimens (figure 1*b*,*c*; electronic supplementary material, S2–S4). This study provides the first observation of *Diplodus annularis* and *Torquigener hypselogeneion* feeding on *D. setosum* (electronic supplementary material, S2). Physical interactions with potentially infected *Diadema* may further contribute to vector transmission, driving the MME on a local scale. In turn, currents and ballast water transfer may serve as transmission vectors on larger regional and global scales, respectively (figure 1*a*). Considering the number of vessels travelling daily through the Suez Canal [46] and the close geographical proximity between the RS and Mediterranean, concerns arise regarding the potential transmission of a pathogen to the native *D. setosum* populations in the RS.

**Table 2.** List of Mediterranean (indigenous and invasive) fish species interacting with *Diadema setosum*. Int.: interaction type (F = feeding, S = shelter), family and species names, origin, feeding strategy and observation localities. Number in brackets refers to counts of specimens observed feeding on dead/dying *D. setosum* (test and spines). Data extracted from electronic supplementary material, S2–S4.

Int.	family	species	origin	feeding strategy
F	Sparidae	*Diplodus vulgaris* (Geoffroy Saint-Hilaire, 1817) (2)	local	carnivore (invertebrates)
F	Sparidae	*Diplodus annularis* (Linnaeus, 1758) (3)	local	carnivore (invertebrates)
F	Sparidae	*Diplodus sargus* (Linnaeus, 1758)	local	carnivore (invertebrates)
F	Scaridae	*Sparisoma cretense* (Linnaeus, 1758) (3)	local	mainly herbivore + small invertebrates
F/S	Labridae	*Thalassoma pavo* (Linnaeus, 1758) (8)	local	carnivore (invertebrates)
F/S	Labridae	*Coris julis* (Linnaeus, 1758)	local	carnivore (invertebrates)
F/S	Siganidae	*Siganus rivulatus* Forsskål & Niebuhr, 1775 (1)	exotic	mainly herbivore
F	Tetraodontidae	*Torquigener hypselogeneion* (Bleeker, 1852) (2)	exotic	carnivore (invertebrates)

## 4. Conclusion

Superficially, mass mortalities of non-native species may seem beneficial, as at least temporarily, they diminish the alien population. However, the currently reported MME of *D. setosum* in the Mediterranean Sea is alarming. Spread of potential pathogens through local and regional processes bears the risk of transfer to native fauna. Furthermore, due to the geographical proximity of the Levantine Basin to the RS, the risk of pathogen transfer to the latter, the native range of *D. setosum*, is imminent—with potential catastrophic consequences. We call for immediate, regional-scale collaboration to facilitate rapid identification of the suspected pathogen and provide real-time monitoring of *D. setosum* in the Mediterranean. These data are crucial to inform local governments and stakeholders of the spread and additional signs of mortalities of this keystone species.

## Data Availability

All additional data are provided in the electronic supplementary material [47].
